# The relationship between markers of antenatal iron stores and birth outcomes differs by malaria prevention regimen—a prospective cohort study

**DOI:** 10.1186/s12916-021-02114-1

**Published:** 2021-10-05

**Authors:** Holger W. Unger, Valentina Laurita Longo, Andie Bleicher, Maria Ome-Kaius, Stephan Karl, Julie A. Simpson, Amalia Karahalios, Elizabeth H. Aitken, Stephen J. Rogerson

**Affiliations:** 1 Department of Obstetrics and Gynaecology, Royal Darwin Hospital, Darwin, Northern Territory Australia; 2 Menzies School of Health Research, Charles Darwin University, Darwin, Northern Territory Australia; 3 Department of Clinical Sciences, Liverpool School of Tropical Medicine, Liverpool, UK; 4 Catholic University of Sacred Heart, Rome, Italy; 5 Department of Obstetrics and Gynaecology, San Pietro-Fatebenefratelli Hospital, Rome, Italy; 6 Department of Medicine (RMH), Peter Doherty Institute for Infection and Immunity, The University of Melbourne, Melbourne, Victoria Australia; 7 Papua New Guinea Institute of Medical Research, Goroka, Papua New Guinea; 8 Australian Institute of Tropical Health & Medicine, James Cook University, Cairns, Australia; 9 Centre for Epidemiology and Biostatistics, Melbourne School of Population and Global Health, University of Melbourne, Melbourne, Victoria Australia; 10 Department of Infectious Diseases, Peter Doherty Institute for Infection and Immunity, The University of Melbourne, Melbourne, Victoria Australia; 11 Department of Microbiology and Immunology, Peter Doherty Institute for Infection and Immunity, The University of Melbourne, Melbourne, Victoria Australia

**Keywords:** Adverse birth outcomes, Iron stores, Iron deficiency, Iron supplementation, Infection, Intermittent preventive treatment, *Plasmodium falciparum*

## Abstract

**Background:**

Iron deficiency (ID) has been associated with adverse pregnancy outcomes, maternal anaemia, and altered susceptibility to infection. In Papua New Guinea (PNG), monthly treatment with sulphadoxine-pyrimethamine plus azithromycin (SPAZ) prevented low birthweight (LBW; <2500 g) through a combination of anti-malarial and non-malarial effects when compared to a single treatment with SP plus chloroquine (SPCQ) at first antenatal visit. We assessed the relationship between ID and adverse birth outcomes in women receiving SPAZ or SPCQ, and the mediating effects of malaria infection and haemoglobin levels during pregnancy.

**Methods:**

Plasma ferritin levels measured at antenatal enrolment in a cohort of 1892 women were adjusted for concomitant inflammation using C-reactive protein and α-1-acid glycoprotein. Associations of ID (defined as ferritin <15 μg/L) or ferritin levels with birth outcomes (birthweight, LBW, preterm birth, small-for-gestational-age birthweight [SGA]) were determined using linear or logistic regression analysis, as appropriate. Mediation analysis assessed the degree of mediation of ID-birth outcome relationships by malaria infection or haemoglobin levels.

**Results:**

At first antenatal visit (median gestational age, 22 weeks), 1256 women (66.4%) had ID. Overall, ID or ferritin levels at first antenatal visit were not associated with birth outcomes. There was effect modification by treatment arm. Amongst SPCQ recipients, ID was associated with a 81-g higher mean birthweight (95% confidence interval [CI] 10, 152; *P* = 0.025), and a twofold increase in ferritin levels was associated with increased odds of SGA (adjusted odds ratio [aOR] 1.25; 95% CI 1.06, 1.46; *P* = 0.007). By contrast, amongst SPAZ recipients, a twofold increase in ferritin was associated with reduced odds of LBW (aOR 0.80; 95% CI 0.67, 0.94; *P* = 0.009). Mediation analyses suggested that malaria infection or haemoglobin levels during pregnancy do not substantially mediate the association of ID with birth outcomes amongst SPCQ recipients.

**Conclusions:**

Improved antenatal iron stores do not confer a benefit for the prevention of adverse birth outcomes in the context of malaria chemoprevention strategies that lack the non-malarial properties of monthly SPAZ. Research to determine the mechanisms by which ID protects from suboptimal foetal growth is needed to guide the design of new malaria prevention strategies and to inform iron supplementation policy in malaria-endemic settings.

**Trial registration:**

ClinicalTrials.gov NCT01136850.

**Supplementary Information:**

The online version contains supplementary material available at 10.1186/s12916-021-02114-1.

## Background

Iron deficiency (ID) is the commonest nutritional deficiency worldwide [1]. In 2017, it ranked fifth in the causes of disability-adjusted-life-years in females [2]. Nearly 40% of pregnant women suffer from anaemia in pregnancy, and ID is the principal cause [3]. Women with ID anaemia have a higher risk of maternal morbidity and mortality due to postpartum haemorrhage and heart failure [4].

ID has been associated with adverse pregnancy outcomes including low birthweight (LBW, birthweight <2500g), preterm birth (PTB, birth before 37 gestational weeks), babies measuring small-for-gestational-age birthweight (SGA), and stillbirth [5, 6]. Infants of iron-deficient mothers are more likely to experience short- and long-term adverse health outcomes, including impacts on neurodevelopment and behaviour [7, 8].

Daily iron supplementation with 30–60 mg of iron plus 400 μg of folic acid is currently recommended for all pregnant women [9]. In sub-Saharan African countries with *Plasmodium falciparum* (*P. falciparum*) transmission, women are additionally advised to use insecticide-treated bed nets, take monthly intermittent preventive treatment (IPTp) with sulphadoxine-pyrimethamine (SP), and seek prompt diagnosis and treatment when experiencing malaria symptoms [9]. Iron supplementation may not be without risk. It has been associated with an increased risk of malaria-associated hospital admissions and deaths amongst children [10], malaria infection during pregnancy [11], and PTB amongst women who took iron supplementation periconceptionally [12].

Measurement of iron stores is essential for planning effective nutritional antenatal interventions and gaining an understanding of the relationship between maternal iron stores and birth outcomes. Serum ferritin reflects the size of total body iron stores and can be used to detect ID, which in pregnant women is defined as a ferritin <15 μg/L [13]. However, ferritin levels are affected by inflammation and infection, including malaria [14]. Consequently, it is recommended to adjust serum ferritin using one or more concurrently measured acute-phase proteins, such as C-reactive protein (CRP) and α-1-acid glycoprotein (AGP) [13, 15, 16].

Anaemia, ID, and malaria are common in pregnant women residing in coastal Papua New Guinea (PNG), a Southwest Pacific island nation with a high burden of adverse maternal and neonatal outcomes [2, 17–19]. A recent, secondary analysis of a PNG pregnancy cohort study reported reduced odds of LBW and PTB, and a higher mean birthweight, in women with ID at antenatal enrolment, in particular in primigravidae [20]. Mediation analysis suggested that this apparently protective effect of ID was largely achieved through mechanisms independent of maternal malaria infection or anaemia. It was hypothesised that ID may confer a level of protection by limiting the proliferation of pathogenic organisms (other than malaria) that are associated with adverse birth outcomes [21]. Others suggested that the observed effect may relate to (unmonitored) iron supplementation in iron-replete women, which could result in blood hyperviscosity and consequential deleterious impacts for foetal growth [6, 22].

Participants in the aforementioned study received a single dose of SP at antenatal enrolment, combined with weekly chloroquine until delivery. A clinical trial subsequently conducted in the same setting compared IPTp with monthly SP plus azithromycin (SPAZ) with a single course of SP plus chloroquine (SPCQ) at antenatal enrolment for the prevention of LBW. IPTp with SPAZ was associated with a marked reduction in LBW risk, via a combination of anti-malarial and unknown non-malarial mechanisms [23]. This finding complements reports of monthly IPTp with SP preventing LBW in women with highly resistant or no *P. falciparum* infection in studies from sub-Saharan Africa [24, 25].

The objective of the present study was to assess the relationship of ID and ferritin levels at first antenatal visit with adverse birth outcomes in women receiving SPAZ or SPCQ and to explore the contributions of malaria infection and haemoglobin (Hb) levels during pregnancy to ferritin-birth outcome relationships through mediation analysis.

## Methods

### Study design

A secondary analysis was conducted of data and samples from a large randomised controlled trial comparing IPTp with SPAZ with a single treatment of SPCQ at antenatal enrolment in PNG [23]. Women followed up for birthweight, and for whom ferritin, Hb, inflammatory markers, and malaria infection status at first antenatal visit could be ascertained, were included in the present analysis.

### Study setting

The study setting is described in detail in the literature [23, 26]. In brief, between November 2009 and February 2013, pregnant women attending one of nine health facilities in Madang Province, PNG, were recruited and followed until birth. Study sites included the Alexishafen health centre, the location of the previous study examining the relationship between antenatal iron status and birthweight in PNG [20]. There is moderate perennial transmission of *P. falciparum* and *P. vivax* in the area, anaemia and ID in pregnancy are common, and more than one-fifth of babies are LBW [20, 27]. At the time of the study, the estimated HIV-1 prevalence at enrolment to antenatal clinics was 1%. SPAZ significantly reduced the risk of LBW (26% relative risk reduction; 95% confidence interval [CI] 9, 40%) and PTB (38%, 95% CI 11–57%) and was associated with a 42 g (95% CI 0, 84 g) higher mean birthweight [23]. SPAZ reduced peripheral malaria parasitaemia and active malarial infection on placental histology, but Hb levels and prevalence of anaemia at birth were similar between trial arms [23].

### Participants

Women were enrolled at 14–26 weeks’ gestation [23]. Screening exclusion criteria for the trial included a symphysis pubis fundal height >26 cm, known adverse reaction to study medications, a permanent disability and chronic medical conditions, known multiple pregnancy, aged <16 years, and symptomatic severe anaemia (Hb <60 g/L).

To be included in the present study, women had to have completed follow-up to delivery. This included delivery of a singleton, congenitally normal live baby with a measured birthweight using electronic scales (Cupid 1, Charder Medical, Taiwan, accuracy of 10 g) [23]. Deliveries <22 gestational weeks were categorised as miscarriages and excluded. All women had a venous blood sample drawn at enrolment and at delivery, peripheral blood smears were prepared, and Hb levels were estimated (HemoCue Ltd, Angelholm, Sweden, accuracy of 1 g/L).

Women were randomised to monthly SP (1500/75 mg) plus AZ (1 g twice daily for 2 days) or a single treatment with SP and chloroquine (CQ, 450 to 600 mg, daily for 3 days) at antenatal enrolment and received a full treatment course at enrolment. All women were also provided with insecticide-treated bed nets. Women with symptomatic malaria infection were treated with quinine (in the first trimester, 300 mg, 2 tablets orally 3 times daily for 7 days) or artemether-lumefantrine (after the first trimester, 20/120 mg, 4 tablets twice daily for 3 days) [23].

Women were provided with iron-folate supplementation (one tablet of ferrous sulphate 270 mg [87.4 mg elemental iron] plus 400 μg folic acid) and advised to take one tablet daily. Women with a Hb ≤90 g/L were advised to take two iron-folate tablets and were provided with albendazole, and scheduled for repeat Hb assessment at 4 weeks. Iron-folate supplementation was not monitored. Cases of symptomatic anaemia were referred to the Madang Provincial Hospital.

### Laboratory analyses

Ferritin concentrations were measured using a novel in-house enzyme-linked immunosorbent assay (ELISA) that was developed, validated, and published by our group [28]. Acute-phase proteins CRP and AGP were measured in venous blood samples from enrolment using commercially available ELISA kits with reference controls (Human Quantikine ELISA kits; R&D Systems, Minneapolis, MN, USA) [29]. Light microscopy of Giemsa-stained thick and thin peripheral and placental blood smears was used to detect *Plasmodium* spp. infection, and peripheral blood and placental samples were additionally tested for *P. falciparum* and *P. vivax* infection by polymerase chain reaction (PCR) [30, 31]. Histology of placental biopsies was examined, as reported previously [32].

### Exposures and outcome measures

The exposures assessed were ferritin (defined as log base 2 [i.e. log_2_] ferritin) and ID, defined as plasma ferritin <15 μg/L at first antenatal visit [13]. Log_2_ transformation was the most appropriate as it resulted in a normal distribution and a linear relationship between ferritin and birthweight, allowed for comparison with earlier research [20], and was the most easily interpretable transformation.

As ferritin (an acute-phase protein) increases during systemic inflammation [15, 16, 33, 34], levels were adjusted using CRP and AGP as markers of inflammation as recommended by the World Health Organization [35]. To do so the “internal regression correction” approach described by the Biomarkers Reflecting the Inflammation and Nutritional Determinants of Anemia (BRINDA) project was used [16, 35]. Internal reference values, i.e. threshold values above which ferritin adjustments are assumed to be required, were 0.2 mg/L and 87 mg/L for CRP and AGP, respectively. BRINDA-adjusted ferritin levels are presented except where stated otherwise.

The birth outcomes of interest were birthweight, LBW, PTB, and SGA (birthweight <10th centile for babies of the same gestational age using INTERGROWTH-21^st^ standards) [36].

### Statistical analysis

The distribution of key socio-demographic and baseline clinical and laboratory characteristics are presented for those who were ID and not ID at antenatal enrolment.

Associations of ferritin or ID with birthweight, LBW, PTB, and SGA were assessed using multivariable linear and logistic regression models, as appropriate. Multivariable regression models were adjusted for the following variables identified a priori as confounders or prognostic factors of birthweight: gravidity, season, number of antenatal clinic visits, clinic location, MUAC, stunting, ethnicity, bed net use, smoking, betel nut use, and gestational age at ferritin measurement (as estimated by fundal height) [18, 23]. Time interval between birth and birthweight measurement to account for weight loss in breastfed newborns (analyses assessing impact on birth weight only), sex of the newborn, treatment arm (overall analyses only), and gestational age at ferritin measurement to account for physiological depletion of iron stores with advancing gestation, were also included in the multivariable regression models, as appropriate. We assessed whether gravidity and malaria prevention strategy (i.e. trial treatment arm) during pregnancy modified the association between iron stores and the birth outcomes by fitting models with interaction terms between iron status (ferritin levels or ID) and gravidity, and models with interaction terms between iron status (ferritin levels or ID) and trial treatment arm; *P*-values for effect modification were derived from likelihood ratio tests comparing models with and without the interaction terms. Sensitivity analyses were conducted using crude plasma ferritin levels, i.e. ferritin levels unadjusted for concomitant inflammation.

Where associations were observed between iron deficiency at enrolment and the outcomes, birthweight and SGA, within each treatment arm (SPCQ and SPAZ), we performed mediation analyses to estimate marginal indirect effects, that is, the effect of iron deficiency on birth outcomes that is operating through the mediating effects of peripheral malaria at enrolment, haemoglobin level at enrolment, peripheral malaria at delivery, placental malaria at delivery and haemoglobin level at delivery, and the marginal natural direct effects, that is, the part of the effect that remains unexplained by the mediators of interest. The mediation analyses were performed using the *paramed* package in Stata. All mediation analyses were adjusted for the confounders: gravidity, season, number of antenatal visits, clinic location, MUAC, stunting, ethnicity, bed net use, smoking, betel nut use, and gestational age at ferritin measurement (as estimated by fundal height).

Malaria infection was defined as the presence of peripheral infection by light microscopy and/or PCR at enrolment or delivery, and placental malaria infection as past or active infection on placental histology [23].

All statistical analyses were performed using Stata version 16.1 (StataCorp, College Station, TX, USA).

### Ethical considerations

Ethical approval for the study was obtained from the Institutional Review Board of the PNG Institute of Medical Research (0815), the PNG Medical Research Advisory Council (8.01), and the Melbourne Health Human Research Ethics Committee (2008.162). The parent trial was registered with the United States National Institutes of Health Clinical Trials Registry (Clinicaltrials.gov, NCT01136850). Informed written consent was obtained from all women. The study was conducted in accordance with Good Clinical Practice guidelines (ICH GCP E6).

## Results

### Participant characteristics at antenatal enrolment

**Fig. 1:**
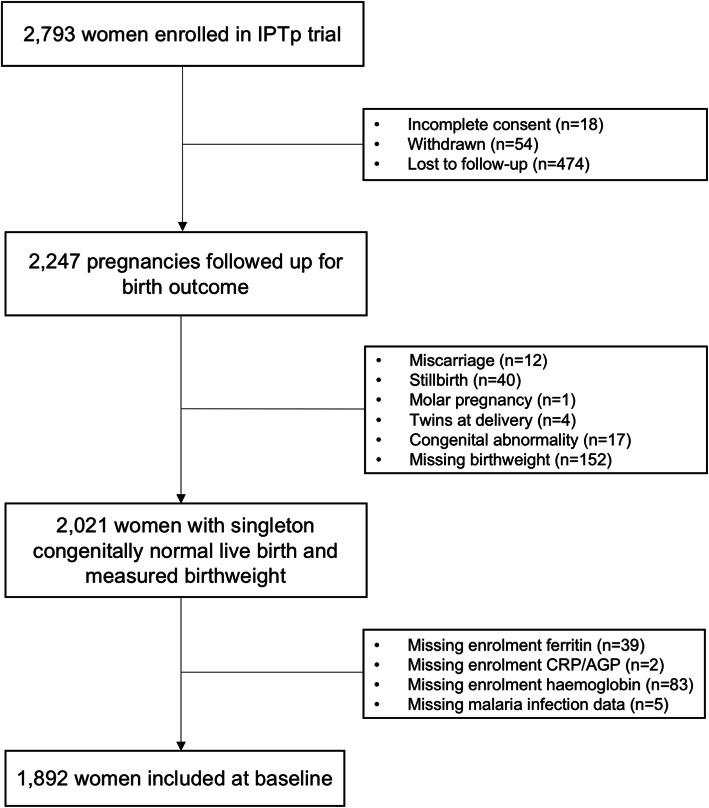
Participant flow chart. Of 2793 women enrolled in the parent trial, 1892 had plasma available for ferritin, alpha-1-acid glycoprotein, and C-reactive protein and were included in the present analyses. Reasons for exclusion are indicated in the diagram. Abbreviations: AGP, α-1-acid glycoprotein; CRP, C-reactive protein; IPTp, intermittent preventive treatment in pregnancy

A total of 1892 women were included in the analysis (Fig. 1). The median age at antenatal enrolment was 23 years (interquartile range [IQR] 20–28 years). Approximately 60% (*n* = 1125) of women resided in rural areas, and the median gestational age at antenatal enrolment was 22 weeks (IQR 19–24 weeks). Nearly one-third of women exhibited signs of macronutrient undernutrition (28.1% with MUAC <23 cm; 18.8% with height <150 cm) and this was the first pregnancy for half of the women (Table 1). A total of 937 (49.5%) and 955 (50.5%) of women received SPCQ and SPAZ, respectively. Three quarters of women (*n* = 1449; 76.6%) reported bed net use during the preceding fortnight.

**Table 1:** Characteristics of women at antenatal enrolment, overall and by presence or absence of iron deficiency. Madang Province, Papua New Guinea, 2009–2013

Characteristic	All women (***N*** = 1892)	Iron-deficient (ferritin <15 μg/L) (***N*** = 1256)	Iron-replete (ferritin ≥15 μg/L) (***N*** = 636)
Age (years)	23 (20, 28)	24 (20, 28)	23 (20, 28)
Smoker			
Yes	362 (19.1)	253 (20.1)	109 (17.1)
No	1528 (80.8)	1001 (79.7)	527 (82.9)
Missing data	2 (0.1)	2 (0.2)	0 (0.0)
Chews betel nut			
Yes	1570 (83.0)	1064 (84.7)	506 (79.6)
No	312 (16.5)	184 (14.7)	128 (20.1)
Missing data	10 (0.5)	8 (0.6)	2 (0.3)
Ethnic origin			
Lowland/Islands	1745 (92.2)	1166 (92.8)	579 (91.0)
Highlands	145 (7.7)	88 (7.0)	57 (9.0)
Missing data	2 (0.1)	2 (0.2)	0 (0.0)
Residence			
Rural	1125 (59.5)	718 (57.2)	407 (64.0)
Urban/peri-urban	759 (40.1)	533 (42.4)	226 (35.5)
Missing data	8 (0.42)	5 (0.4)	3 (0.5)
Clinic			
Alexishafen	326 (17.2)	210 (16.7)	116 (18.2)
Others	1566 (82.8)	1046 (83.3)	520 (81.8)
Gestational age (weeks)^1*^	22 (19, 24)	23 (20, 25)	20 (17, 23)
MUAC (cm)^*^	23.9 [2.6]	23.8 [2.5]	24.1 [2.6]
Height (cm)^*^	154 [6]	154 [6]	155 [6]
BMI (kg/m^2^)^*^	22.5 (20.9, 24.3)	22.5 (20.9, 24.2)	22.5 (20.8, 24.7)
Gravidity			
Primigravid	932 (49.3)	582 (44.3)	350 (55.0)
Multigravid	957 (50.6)	672 (53.5)	285 (44.8)
Missing data	3 (0.2)	2 (0.2)	1 (0.2)
Malaria prevention			
SPAZ	50.5 (955)	629 (50.1)	326 (51.3)
SPCQ	49.5 (937)	627 (49.9)	310 (48.7)
Used bed net during preceding fortnight			
Yes	1449 (76.6)	971 (77.3)	478 (75.2)
No	438 (23.2)	282 (22.5)	156 (24.5)
Missing data	5 (0.3)	3 (0.2)	2 (0.3)
Haemoglobin (g/L)	97 [15]	96 [14]	99 [16]
Anaemia (g/L)			
>100	728 (38.5)	440 (35.0)	288 (45.3)
>90 and ≤100	618 (32.7)	441 (35.1)	177 (27.8)
>70 and ≤90	468 (24.7)	322 (25.6)	146 (23.0)
≤70	78 (4.1)	53 (4.2)	25 (3.9)
Malaria infection^2^			
Present	252 (13.3)	122 (9.7)	130 (20.4)
Absent	1640 (86.7)	1134 (90.3)	506 (79.6)

Note. Mean [standard deviation]; or median (interquartile range); or *n* (%). Ferritin levels were adjusted for concomitant inflammation using the BRINDA (Biomarkers Reflecting Inflammation and Nutritional Determinants of Anemia) approach [16]

*Abbreviations*: *AGP*, α-1-acid glycoprotein; *BMI*, body mass index (kg/m^2^); *CRP*, C-reactive protein; *MUAC*, mid-upper arm circumference. *P. falciparum*, *Plasmodium falciparum*

^1^According to symphysis pubis fundal height in cm at antenatal enrolment

^2^*Plasmodium* (*P*.) *falciparum* and *P. vivax* infection in peripheral blood, as detected by light microscopy and polymerase chain reaction

^*^Gestational age (*n* = 1889), MUAC (*n* = 1852), height (*n* = 1861), and BMI (*n* = 1,856)

The mean ± standard deviation (SD) haemoglobin (Hb) at the antenatal enrolment was 97 g/L ± 15 g/L. A total of 61.5% (*n* = 1164) of women were anaemic (Hb ≤100 g/L, as per PNG guidelines [37]): 32.7% (*n* = 618) had mild anaemia (Hb > 90 and ≤ 100 g/L), 24.7% (468) had moderate anaemia (Hb >70 and ≤90 g/L), and 4.1% (78) had severe anaemia (Hb ≤70 g/L). *Plasmodium* spp. infections were detected in 13.3% of women at antenatal enrolment (*n* = 252) (Table 1): *P. falciparum* was the predominant species (*n* = 184; 9.7%), and half of infections were submicroscopic (*n* = 129; 6.8%).

Ferritin concentrations at antenatal enrolment were low (median 9.8 μg/L; IQR 5.4–19.5 μg/L). Two-thirds of women (*n* = 1256) had ID (ferritin <15 μg/L). Women with ID more frequently chewed areca (betel) nut (84.7% vs. 79.6%), had an urban/peri-urban residence (42.4% vs. 35.5%), and were more commonly multigravid (53.5% vs. 44.8%). Women with ID had a higher median gestational age at enrolment (23; IQR 20–25) compared to iron-replete women (20; IQR 17–23), consistent with gestational depletion of iron stores (Table 1). Women with ID were more likely to be anaemic compared to iron-replete women (65.0% vs. 54.7%). Iron-deficient women were less likely to be malaria-infected at enrolment (9.7% vs. 20.4%). Using unadjusted ferritin levels to estimate ID yielded similar results (Additional file 1: Table S1).

### Birth outcomes

The mean birthweight in the cohort was 2943 g (SD 482 g), and 15.1% of babies (*n* = 285) were LBW. Amongst women with ultrasound-dated pregnancies (*n* = 1243), the median gestational age at birth was 38 weeks (IQR 38–40 weeks); 9.0% (112/1243) and 24.4% (303/1243) of women delivered a PTB and SGA baby, respectively. At delivery, a total of 6.3% (117/1867) of women had peripheral *P. falciparum* (68 mono-infections, three mixed infections with *P. vivax*) or *P. vivax* (*n* = 46): 56 of these infections were submicroscopic (3.0%). Amongst women with histology data, 7.4% (98/1333) had active placental infection, and 11.2% (149/1333) had evidence of past infection. Fifty-five percent of women were anaemic (931/1709) and the mean Hb at birth was 101 g/L (SD 17 g/L).

### Relationship of iron stores at antenatal enrolment with birth outcomes

Results from fitting multivariable models examining the study population as a whole showed that for a twofold increase in ferritin levels, there was a decrease of 11 g (95% CI −27, 6; *P* = 0.19) in mean birthweight (Table 2). Babies of women with ID were on average 46 g (95% CI −0.4, 92 g; *P* = 0.052) heavier compared to iron-replete women (Table 2). Ferritin levels and ID were not associated with LBW (Table 2). Associations of ferritin levels with birthweight appeared to be driven by impacts on foetal growth rather than length of gestation. For a twofold increase in ferritin levels, there were increased odds of SGA (OR 1.11; 95% CI 0.99, 1.24; *P* = 0.066), and ferritin levels or ID were not associated with preterm birth (Table 2). Using unadjusted ferritin levels yielded similar results (see Additional file 1: Table S2 [16, 36]).

**Table 2:** Associations of maternal ferritin at first antenatal visit with birthweight, low birthweight (*n* = 1840), small-for-gestational age, and preterm birth (*n* = 1208), overall and stratified by malaria prevention regimen. Madang Province, Papua New Guinea, 2009–2013

	Overall	Malaria prevention regimen		
	Adjusted mean difference (95% CI); ***P***	SPCQ (***n*** = 898) Adjusted mean difference (95% CI); ***P***	SPAZ (***n*** = 934) Adjusted mean difference (95% CI); ***P***	***P*** interaction parameter
**Birthweight (grammes)**				
*Iron stores (measured by ferritin) (log*_*2*_*) μg/L*	−11 (−27, 6); 0.19	−26 (−50, −1); 0.041	9 (−13, 31); 0.45	0.041
*Iron deficiency*				
Ferritin <15 μg/L	46 (−0.4, 92); 0.052	81 (10, 152); 0.025	6 (−54; 66); 0.845	0.11
Ferritin ≥15 μg/L	Reference	Reference	Reference	
	**Adjusted OR (95% CI);*P***	**Adjusted OR (95% CI);*P***	**Adjusted OR (95% CI);*P***	
**Low birthweight (<2500 g)**				
*Iron stores (measured by ferritin) (log*_*2*_*) μg/L*	1.00 (0.90, 1.11); 0.99	1.15 (1.00, 1.32); 0.050	0.80 (0.67, 0.94); 0.009	0.001
*Iron deficiency*				
Ferritin <15 μg/L	0.86 (0.64, 1.15); 0.31	0.73 (0.49, 1.08); 0.12	1.10 (0.70, 1.73); 0.69	0.18
Ferritin ≥15 μg/L	Reference	Reference	Reference	
**Preterm birth (<37 weeks)**		**SPCQ (*n*= 586)**	**SPAZ (*n*= 622)**	
*Iron stores (measured by ferritin) (log*_*2*_*) μg/L*	1.03 (0.87, 1.20); 0.77	1.03 (0.84, 1.27); 0.79	0.98 (0.75, 1.28); 0.87	0.91
*Iron deficiency*				
Ferritin <15 μg/L	0.85 (0.54, 1.34); 0.48	0.94 (0.51, 1.73); 0.84	0.82 (0.40, 1.65); 0.57	0.62
Ferritin ≥15 μg/L	Reference	Reference	Reference	
**Small-for-gestational-age**^a^				
*Iron stores (measured by ferritin) (log*_*2*_*) μg/L*	1.11 (0.99, 1.24); 0.066	1.25 (1.06, 1.46); 0.007	0.98 (0.84, 1.15); 0.80	0.02
*Iron deficiency*				
Ferritin <15 μg/L	0.79 (0.59, 1.07); 0.12	0.67 (0.44, 1.04); 0.072	0.91 (0.60, 1.40); 0.77	0.19
Ferritin ≥15 μg/L	Reference	Reference	Reference	

Note. Linear regression analyses were performed for the outcome birthweight, with mean difference (95% CI) presented and logistic regression for the outcomes, low birthweight, preterm birth, and small-for-gestational age, with odds ratios (95% CI) presented. Analyses were adjusted for gravidity, season, number of antenatal visits, clinic location, mid-upper arm circumference (< 23cm, ≥ 23 cm), stunting (height <150 cm, ≥ 150 cm), ethnicity, bed net use, smoking, betel nut use, and gestational age at enrolment, i.e. at ferritin measurement (as estimated by fundal height). Models also included the covariates sex of the newborn, timing of birthweight measurement (analyses including birthweight only), and treatment arm (overall analysis only). Ferritin levels were adjusted for concomitant inflammation using the BRINDA (Biomarkers Reflecting Inflammation and Nutritional Determinants of Anemia) approach [16]

*Abbreviations*: *CI*, confidence interval; *OR*, odds ratio; *SPAZ*, sulphadoxine-pyrimethamine plus azithromycin; *SPCQ*, SP plus chloroquine

^a^Defined as birthweight <10th centile of the Intergrowth-21 reference [36]

### Impact of malaria chemoprevention during pregnancy on the relationship between antenatal iron status and birth outcomes

Associations between ferritin levels at antenatal enrolment and birthweight differed between women who were treated with monthly SPAZ and those treated with a single course of SPCQ (*P* = 0.041 for birthweight, *P* = 0.001 for LBW, and *P* = 0.02 for SGA; *P*-values for interaction terms) (Table 2). Amongst SPCQ recipients, for a twofold increase in ferritin levels, there was a decrease of 26 g (95% CI −50, −1; *P* = 0.041) in mean birthweight, increased odds of LBW (aOR 1.15; 95% CI 1.00, 1.32; *P* = 0.050), and increased odds of SGA (aOR 1.25; 95% CI 1.06, 1.46; *P* = 0.007). Conversely, in the SPAZ group, for a twofold increase in ferritin levels, there was an increase of 9 g (95% CI −13, 31; *P* = 0.45) in mean birthweight, reduced odds of LBW (OR 0.80; 0.67, 0.94; *P* = 0.009), and no difference in the odds of SGA (aOR 0.98; 95% CI 0.84, 1.15; *P* = 0.80) (Table 2). Associations between ferritin levels or ID with PTB were not modified by malaria prevention regimen (*P* =0.91 for ferritin and *P* = 0.62 for ID; *P* values for interaction terms). Analogous associations were observed when ID was the exposure of interest (Table 2). In women receiving SPCQ, ID was associated with an increase of 81 g (95% CI 10, 152; *P* = 0.025) in mean birthweight and reduced odds of LBW (aOR 0.73; 95% CI 0.49, 1.08; *P* = 0.12) or SGA (aOR 0.67; 95% CI 0.44, 1.04; *P* = 0.072). ID was not associated with birthweight, LBW, SGA, or preterm birth in women randomised to SPAZ (all *P* > 0.1). Using unadjusted ferritin levels yielded similar results (Additional file 1: Table S2 [16, 36]). When we stratified the relationship between iron deficiency and pregnancy outcomes by gravidity, there were tendencies towards stronger associations in primigravid than multigravid women, but these were not statistically significant (Additional file 1: Table S3 [16, 36]).

### Mediation of iron-birth outcome relationships by malaria infection and haemoglobin in SPCQ recipients

**Fig. 2:**
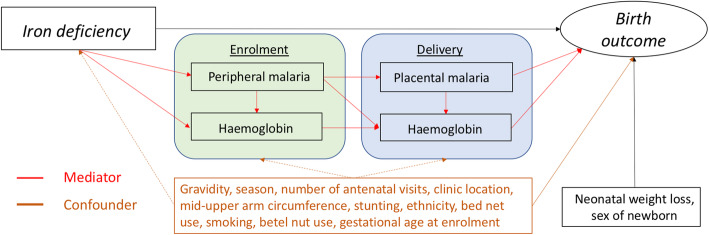
Conceptual directed acyclic graph of mediation iron deficiency (ferritin <15 μg/L) and birthweight by malaria infection and maternal haemoglobin. Peripheral malaria was defined as the presence of *P. falciparum and/or P. vivax* infections on light microscopy and/or polymerase chain reaction, and placental malaria as active or past infection on histology

Mediation analysis was conducted to determine whether, amongst SPCQ recipients, associations of ID with birthweight or SGA are mediated by malaria infection or haemoglobin levels during pregnancy and at birth (Fig. 2, Table 3). There were protective effects of ID on birthweight that were not mediated by malaria infection (natural direct effect) (88.9 g; 95% CI 17.2, 160.8 g) or haemoglobin at enrolment (78.9 g; 95% CI 7.8, 150.0 g), with analogous directionality (not statistically significant) observed when assessing for mediation by malaria infection status (peripheral or placental infection) or haemoglobin at birth (Table 3). At the most, 13% (6.1 g of a total protective effect of 46.8 g) of the association between ID and higher mean birthweight was mediated through pathways that included placental infection (Table 3). ID conferred a direct protective effect on the odds of SGA not mediated by malaria infection (aOR 0.62; 95% CI 0.40, 0.95) or haemoglobin at enrolment (aOR 0.65; 95% CI 0.42, 0.99), and similar trends were observed in models examining indirect pathways via malaria infection or anaemia at birth (Table 3).

**Table 3:** Mediation of the association between iron deficiency (ferritin <15 μg/L) at enrolment and the birth outcomes birthweight and SGA for pregnant women in the SPCQ treatment group. Madang Province, Papua New Guinea, 2009–2013

	Mediator				
	Peripheral malaria at enrolment	Haemoglobin at enrolment	Peripheral malaria at delivery	Placental malaria at delivery	Haemoglobin at delivery
**Outcome—birthweight (grammes)**	Mean difference (95% CI); *n* = 901	Mean difference (95% CI); *n* = 901	Mean difference (95% CI); *n* = 893	Mean difference (95% CI); *n* = 747	Mean difference (95% CI); *n* = 815
Total effect	79.0 (1.0, 157.1)	78.6 (7.5, 149.7)	68.3 (−5.5, 142.1)	46.8 (−35.6, 129.2)	58.7 (−15.5, 132.9)
Natural direct effect	88.9 (17.2, 160.8)	78.9 (7.8, 150.0)	67.6 (−2.9, 138.0)	40.7 (−39.1, 120.6)	57.2 (−17.3, 131.7)
Indirect effect	−9.9 (−21.5, 1.6)	−0.3 (−2.0, 1.5)	0.7 (−1.7, 3.1)	6.1 (−3.3, 15.4)	1.5 (−5.8, 8.8)
**Outcome—small for gestational age**^a^	OR (95% CI); *n* = 589	OR (95% CI); *n* = 589	OR (95% CI); *n* = 583	OR (95% CI); *n* = 486	OR (95% CI); *n* = 538
Total effect	0.64 (0.41, 1.00)	0.65 (0.42, 0.99)	0.65 (0.42, 1.00)	0.71 (0.43, 1.17)	0.56 (0.36, 0.89)
Natural direct effect	0.62 (0.40, 0.95)	0.65 (0.42, 0.99)	0.66 (0.43, 1.02)	0.71 (0.44, 1.15)	0.59 (0.37, 0.93)
Indirect effect	1.04 (0.93, 1.16)	1.00 (0.99, 1.01)	0.98 (0.90, 1.06)	1.00 (0.86, 1.17)	0.96 (0.89, 1.03)

Note. Analyses adjusted for the confounders: gravidity, season, clinic location, mid-upper arm circumference (< 23cm, ≥ 23 cm), stunting (height <150 cm, ≥ 150 cm), ethnicity, bed net use, smoking, betel nut use, and gestational age at enrolment, i.e. at ferritin measurement (as estimated by fundal height). Ferritin levels were adjusted for concomitant inflammation using the BRINDA (Biomarkers Reflecting Inflammation and Nutritional Determinants of Anemia) approach [16]

*Abbreviations*: *CI*, confidence interval; *OR*, odds ratio; *SPCQ*, sulphadoxine-pyrimethamine plus chloroquine

^a^Defined as birthweight <10^th^ centile of the Intergrowth-21 reference [36]

## Discussion

In a large pregnancy cohort in PNG, nearly two-thirds of women were found to be iron deficient at antenatal enrolment. In analyses considering all women, there was a decrease in mean birthweight and increased odds of SGA associated with twofold increases in mid-pregnancy ferritin levels (not statistically significant), with reciprocal increases in mean birthweight and reduction in the odds of SGA amongst iron-deficient women. Associations between measures of antenatal iron stores and the birth outcomes birthweight, LBW, and SGA were altered by the type of malaria chemoprevention during pregnancy, but not by gravidity. Amongst women who received SPCQ, ID was associated with increased mean birthweight and lower odds of SGA; this was mirrored by increased odds of SGA associated with a twofold increase in ferritin. Conversely, in SPAZ recipients, a twofold increase in ferritin was associated with lower odds of LBW. Ferritin and ID were not associated with PTB. Malaria infection during pregnancy and Hb did not mediate associations between ID and birthweight or SGA amongst SPCQ recipients.

Maternal ID and associated iron-deficiency anaemia have been associated with adverse birth outcomes including LBW, PTB, and SGA [38–40]. Meta-analysis suggests that restoring or maintaining adequate maternal iron stores through iron supplementation prevents adverse pregnancy outcomes [3]. Benefits of iron supplementation for the prevention of adverse pregnancy outcomes may be more pronounced in settings with a higher prevalence of ID [41]. The WHO thus recommends universal iron supplementation (and augmented iron supplementation in anaemic women), one of several key components of antenatal care globally [9]. The WHO further recommends contemporaneous prevention and treatment of endemic infections such as malaria and hookworm, as they have been associated with anaemia through blood loss, increased erythrocyte turnover, and reduced red cell production [9]. However, studies assessing relationships between markers of maternal iron reserves and birth outcomes do not consistently report a reduction in adverse outcomes in iron-replete women [42, 43]. Recent studies report possible associations of ID during pregnancy with reduced risks of adverse pregnancy outcomes and increases in mean birthweight, as well as associations of iron supplementation during pregnancy with an increased risk of LBW [20, 44–48].

Several mechanisms to explain the association between an iron-replete state with reductions in birthweight have been proposed [20–22, 45]. Iron deficiency may lead to decreased reticulocyte numbers, and thus in malaria-endemic settings such as PNG, ID may confer a level of protection from *P. falciparum* infection. This is due to invasion of, and proliferation in, older red blood cells being poor, whilst supplementation may result in reticulocytosis, increasing numbers of available young erythrocytes [49–53]. ID was associated with reduced malaria risk in pregnant women in a 2014 meta-analysis, and in one study, iron supplementation in pregnancy was associated with an increased risk of *P. vivax* infection [11]. In the earlier PNG cohort study, ID was not associated with malaria risk, and modelling suggested a substantial direct “protective” effect of ID on the risk of low birthweight (risk ratio 0.44; 95 CI 0.25, 0.79) that was not mediated through protection against peripheral malaria at enrolment, anaemia, and placental malaria [20]. The authors hypothesised that an iron-deficient state may confer protection against infectious pathogens other than malaria that are associated with reduced birthweight [21], given the crucial role iron plays in their metabolism and survival [54, 55].

Women in the present study were randomised to two markedly different malaria prevention strategies, and ferritin-birth outcome relationships differed between trial arms. Women either received a single malaria clearance treatment with SPCQ at antenatal enrolment (reflecting previous PNG malaria policy) or intermittent preventive treatment (IPTp) with monthly SPAZ. Monthly IPTp prevents LBW even in women without malaria or in settings where SP has lost its anti-malarial efficacy due to drug resistance [24, 56, 57]. These non-malarial benefits of SP, which are substantial and may relate to the prevention of other infections, could be of importance. Broad antimicrobial properties of SP, or of SPAZ, could abrogate the putative risk that iron supplementation (or an iron-replete state) may pose for infections that cause adverse birth outcomes. SPAZ was found to nullify the association between inflammation (as measured by CRP and AGP) and adverse birth outcomes [29]. Non-malarial benefits of SP may explain why in clinical trials of iron supplementation conducted in Kenya and Tanzania iron supplementation improved birthweight, as supplementation was given alongside IPTp with SP [41, 58]. Similarly, higher mid-trimester ferritin levels were associated with reduced odds of LBW in women receiving IPTp with SPAZ in the present study. In settings with high burdens of infectious pathogens and a high burden of iron deficiency, iron supplementation is safe and appears to translate into improvements in birth outcomes when given in combination with SP or SPAZ.

Furthermore, it has been proposed that the observed ferritin-birth outcome relationship may be due to an adverse effect of iron supplementation on birthweight in iron-replete women, amongst whom supplementation may reduce birthweight [6, 22, 45]. Putative mechanisms may include adverse impacts on foetal growth due to either increased blood viscosity or placental damage from free radicals in supplemented iron-replete women [59, 60]. Amongst pregnant Gambian women receiving IPTp with SP, limiting iron supplementation to women in whom iron-deficiency anaemia was detected through a hepcidin-guided screen-and-treat intervention had no advantage over universal supplementation in terms of adherence, side-effects, or safety outcomes [61]. Mediation analyses in the present and prior study from PNG suggest that the protective effect of ID is largely achieved through mechanisms independent of maternal malaria infection or anaemia. Furthermore, delivery haemoglobin levels did not differ between trial arms [23], and few women in our cohort had haemoglobin levels >130 g/L (enrolment 1.3%, 25/1892; delivery 4.2%, 71/1709). Haemoglobin concentrations above 130 g/L may be associated with increased odds of SGA [6]. Assuming a similar intake of iron supplements across both arms following randomisation, the observed effects are unlikely to be mediated by impacts of iron supplementation on increased blood viscosity and possible adverse consequences for foetal growth that may be associated with it.

The relationship between maternal iron status or iron supplementation and birth outcomes should also be a consideration in research evaluating other candidate regimens for IPTp. Combinations such as dihydroartemisinin-piperaquine (DP) have superior anti-malarial efficacy compared to SP, yet lack the non-malarial benefits of SP for the prevention of adverse birth outcomes [25]. Causal mediation analysis of three clinical trials comparing both regimens demonstrated that the mean birthweight amongst SP recipients was approximately 70 g higher compared to DP recipients. SP conferred a greater non-malarial effect than DP (87 g difference in mean birthweight), whereas DP conferred a larger anti-malarial effect compared to SP (8 to 31 g difference in mean birthweight, depending on dosing frequency) [25]. Further research needs to determine how SP or SPAZ alter the relationship between maternal iron status or iron supplementation and birth outcomes. The impact (and its directionality) of IPTp with DP on relationships of maternal iron status or supplementation with birth outcomes is unknown. Our research suggests that the evaluation of alternative IPT regimens should integrate assessments of the impact of maternal iron status or iron supplementation on birth outcomes, as compared to SP. In the absence of suitable alternatives, efforts to upscale the roll-out of IPTp with SP, to be provided alongside iron supplementation, seem crucial.

Our study replicates only some of the findings of a prior investigation, at one of our study sites [20]. In 279 women receiving SPCQ and weekly CQ prophylaxis, those with ID had a significantly higher mean birthweight (230 g) and reduced odds of LBW (aOR 0.32) and PTB (aOR 0.57) [20]. ID was more common in women included in the previous study (71%, estimated using unadjusted ferritin levels) compared to the present study (39.4%, based on unadjusted ferritin, Supplemental table 1). Greatest effects were seen amongst primigravidae. In our larger study over a wider geographical catchment area including rural and urban residents, we found lower burdens of malaria infection and anaemia. ID was associated with a modest increase in mean birthweight in women randomised to a single dose of SPCQ, and gravidity did not modify the iron-birthweight relationship. Importantly, we used foetal biometry to estimate gestational age in two-thirds of women, which adds crucial precision in the estimation of PTB and SGA burdens [62]. Our study indicates that ferritin-birthweight relationships may be driven by impacts on foetal growth rather than preterm birth.

Sample size, measurement of two markers of inflammation to adjust ferritin levels, and assessment of malaria infection by qPCR and placental histology are important strengths of the present study. Furthermore, the design of the parent trial permitted an evaluation of the impact of different pharmacological malaria prevention strategies on the relationship between iron status and birth outcomes. Limitations include the lack of assessment of the presence of other infections, including chorioamnionitis and genitourinary tract infections. Furthermore, iron supplementation following enrolment was unsupervised and uptake may have differed between trial arms. We only assessed foetal biometry and placental infection in a proportion of women and only evaluated ferritin-birth outcome relationships in women with live births, who completed follow-up for birthweight and for whom measures of malaria and anaemia were available [23]. As previously reported, women excluded from birthweight analyses were more commonly malaria infected at antenatal enrolment, more commonly resided in rural areas, more likely to be illiterate, but did not differ in other characteristics, including MUAC and enrolment haemoglobin [23]. We performed multiple comparisons in this exploratory analysis, and some associations may be due to chance and must be interpreted with caution [63]. Lastly, estimation of iron status using ferritin, even when adjusted for concomitant inflammation, is likely to be less precise compared to measures such as transferrin receptor/log_10_ ferritin ratio or hepcidin [64]. Residual confounding, potentially due to unadjusted inflammation/infection may thus drive some of the effects observed.

## Conclusions

In coastal PNG, ID at first antenatal visit is common. The relationship between maternal iron status and adverse birth outcomes is altered by the type of malaria chemoprevention strategy that is offered during pregnancy. Improved antenatal iron stores are beneficial for LBW prevention in women receiving monthly SPAZ. They do not confer a benefit for the prevention of adverse birth outcomes amongst SPCQ recipients, amongst whom an iron-replete state may hinder optimal foetal growth through processes that are largely independent of malaria infection and maternal haemoglobin. Research to determine the mechanisms by which ID protects from suboptimal foetal growth is needed to guide the design of new malaria prevention strategies and to inform iron supplementation policy in malaria-endemic settings.

## Supplementary Information

**Additional file 1: Tab S1**. Characteristics of participants by unadjusted ferritin levels. **Tab S2**. Unadjusted ferritin and pregnancy outcomes. **Tab S3**. Maternal iron status and pregnancy outcomes, stratified by gravidity.

## Data Availability

Data are available from the WWARN data repository (http://www.wwarn.org/working-together/sharing-data/accessing-data) for researchers who meet the criteria for access to confidential data and from the corresponding author on reasonable request.

## Abbreviations

AGP: α-1-Acid glycoprotein
CI: Confidence interval
CRP: C-reactive protein
DP: Dihydroartemisinin-piperaquine
ELISA: Enzyme-linked immunosorbent assay
Hb: Haemoglobin
ID: Iron deficiency
IPTp: Intermittent preventive treatment in pregnancy
IQR: Interquartile range
LBW: Low birthweight
LMIC: Low- and middle-income countries
MD: Mean difference
MUAC: Mid-upper arm circumference
OR: Odds ratio
PNG: Papua New Guinea
PTB: Preterm birth
qPCR: Quantitative real-time polymerase chain reaction
SD: Standard deviation
SGA: Small-for-gestational-age
SP: Sulphadoxine-pyrimethamine
SPAZ: Sulphadoxine-pyrimethamine plus azithromycin
SPCQ: Sulphadoxine-pyrimethamine plus chloroquine
