# Supplementary material for: The relationship between markers of antenatal iron stores and birth outcomes differs by malaria prevention regimen—a prospective cohort study
Source: BMC Med. 2021 Oct 5;19:236. doi: 10.1186/s12916-021-02114-1 (PMC8491429; doi:10.1186/s12916-021-02114-1)
Supplement: Supplementary file 1 — Additional file 1: Tab S1. Characteristics of participants by unadjusted ferritin levels. Tab S2. Unadjusted ferritin and pregnancy outcomes. Tab S3. Maternal iron status and pregnancy outcomes, stratified by gravidity. [file 12916_2021_2114_MOESM1_ESM.docx]

**Table S1** Characteristics of women at antenatal enrolment, overall and by presence or absence of iron deficiency (crude plasma ferritin). Madang Province, Papua New Guinea, 2009-2013.

| **Characteristic** | | **All women**  **(N = 1892)** |  | **Iron-deficient**  **(ferritin <15 μg/L)**  **(N=745)** | **Iron-replete**  **(ferritin ≥15 μg/L) (N=1,147)** |
| --- | --- | --- | --- | --- | --- |
| Age (years) | | 23 (20, 28) |  | 24 (20, 28) | 23 (20, 28) |
| Smoker | |  |  |  |  |
|  | Yes | 362 (19.1) |  | 158 (21.2) | 204 (17.8) |
|  | No | 1,528 (80.8) |  | 586 (78.7) | 942 (82.1) |
|  | Missing data | 2 (0.1) |  | 1 (0.1) | 1 (0.1) |
| Chews betel nut | |  |  |  |  |
|  | Yes | 1,570 (83.0) |  | 639 (85.8) | 931 (81.2) |
|  | No | 312 (16.5) |  | 103 (13.8) | 209 (18.2) |
|  | Missing data | 10 (0.5) |  | 3 (0.4) | 7 (0.6) |
| Ethnic origin | |  |  |  |  |
|  | Lowland/Islands | 1,745 (92.2) |  | 684 (91.8) | 1,061 (92.5) |
|  | Highlands | 145 (7.7) |  | 60 (8.1) | 85 (7.4) |
|  | Missing data | 2 (0.1) |  | 1 (0.1) | 1 (0.1) |
| Residence | |  |  |  |  |
|  | Rural | 1,125 (59.5) |  | 395 (52.0) | 730 (63.6) |
|  | Urban/peri-urban | 759 (40.1) |  | 347 (46.6) | 412 (35.9) |
|  | Missing data | 8 (0.42) |  | 3 (0.4) | 5 (0.4) |
| Clinic | |  |  |  |  |
|  | Alexishafen | 326 (17.2) |  | 111 (14.9) | 215 (18.7) |
|  | Other | 1,566 (82.8) |  | 634 (85.1) | 932 (81.3) |
| Gestational age (weeks)^1*^ | | 22 (19, 24) |  | 23 (21, 25) | 21 (18, 24) |
| MUAC (cm)^*^ | | 23.9 [2.6] |  | 23.8 [2.5] | 24.0 [2.6] |
| Height (cm)^*^ | | 154 [6] |  | 154 [6] | 154 [6] |
| BMI (kg/m^2^)^*^ | | 22.5 (20.9, 24.3) |  | 22.4 (21.0, 24.1) | 22.5 (20.8, 24.4) |
| Gravidity | |  |  |  |  |
|  | Primigravid | 932 (49.3) |  | 328 (44.0) | 604 (52.7) |
|  | Multigravid | 957 (50.6) |  | 416 (55.8) | 541 (47.2) |
|  | Missing data | 3 (0.2) |  | 1 (0.1) | 2 (0.2) |
| Malaria prevention | |  |  |  |  |
|  | SPAZ | 50.5 (955) |  | 364 (48.9) | 591 (51.5) |
|  | SPCQ | 49.5 (937) |  | 381 (51.1) | 556 (48.5) |
| Used bed net during preceding fortnight | |  |  |  |  |
|  | Yes | 1,449 (76.6) |  | 577 (77.5) | 872 (76.0) |
|  | No | 438 (23.2) |  | 166 (22.3) | 272 (23.7) |
|  | Missing data | 5 (0.3) |  | 2 (0.3) | 3 (0.3) |
| Haemoglobin (g/L) | | 97 [15] |  | 95 [14] | 99 [15] |
| Anaemia (g/L) | |  |  |  |  |
|  | >100 | 728 (38.5) |  | 236 (31.7) | 492 (42.9) |
|  | >90 & ≤100 | 618 (32.7) |  | 262 (35.2) | 356 (31.0) |
|  | >80 & ≤90 | 468 (24.7) |  | 205 (27.5) | 263 (22.9) |
|  | ≤70 | 78 (4.1) |  | 42 (5.6) | 36 (3.1) |
| CRP (mg/L) | | 1.4 (0.5, 3.7) |  | 1.2 (0.5, 2.7) | 1.6 (0.6, 4.3) |
|  | ≤5 | 1,543 (81.6) |  | 657 (88.2) | 886 (77.2) |
|  | >5 | 349 (18.5) |  | 88 (11.8) | 261 (22.8) |
| AGP (mg/L) | | 230 (145, 362) |  | 210 (138, 337) | 245 (153, 377) |
|  | ≤500 | 1,641 (86.7) |  | 664 (89.1) | 977 (85.2) |
|  | >500 | 251 (13.3) |  | 81 (10.9) | 170 (14.8) |
| Malaria infection^2^ | |  |  |  |  |
|  | Present | 252 (13.3) |  | 83 (8.5) | 189 (16.5) |
|  | Absent | 1,640 (86.7) |  | 682 (91.5) | 958 (83.5) |

**Note.** Mean [standard deviation]; or median (interquartile range); or N (%).

Abbreviations: AGP, α-1-acid glycoprotein; BMI, body mass index (kg/m^2^); CRP, C-reactive protein; MUAC, mid-upper arm circumference. *P. falciparum*, *Plasmodium falciparum*.

^1^ According to symphysis-pubis fundal height in cm at antenatal enrolment

^2^ *Plasmodium (P.) falciparum*, *P. vivax, P. malariae, P. oval infection in peripheral blood, as detected by polymerase chain reaction*

^*^ Gestational age (n=1,889), MUAC (n=1,852), height (n=1,861), BMI (n=1,856).

**Table S2** Associations of maternal iron status (unadjusted ferritin) at first antenatal visit with birthweight, low birthweight (n=1,832), fetal growth restriction, and preterm birth (n=1,208), overall and stratified by malaria prevention regimen. Madang Province, Papua New Guinea, 2009-2013.

|  | **Overall** | **Malaria prevention regimen** | |  |
| --- | --- | --- | --- | --- |
|  | **Adjusted mean difference, grams (95% CI); *p*** | **SPCQ (n=898)**  **Adjusted mean difference, grams (95% CI); *p*** | **SPAZ (n=934)**  **Adjusted mean difference, grams (95% CI); *p*** | ***p* interaction parameter** |
| **Birthweight** |  |  |  |  |
| *Iron stores (measured by ferritin)* *(log_2_) μg/L* | -13 (-29, 8); 0.13 | -27 (-51, -2); 0.033 | 6 (-16, 28); 0.58 | 0.046 |
|  |  |  |  |  |
| *Iron deficiency* |  |  |  |  |
| Ferritin <15 μg/L | 13 (-31, 58); 0.56 | 24 (-34, 101); 0.32 | -13 (-72, 46); 0.66 | 0.27 |
| Ferritin ≥15 μg/L | Reference | Reference | Reference |  |
|  |  |  |  |  |
| **Low birthweight (<2,500 g)** | **Adjusted OR (95% CI); *p*** | **Adjusted OR (95% CI); *p*** | **Adjusted OR (95% CI); *p*** |  |
| *Iron stores (measured by ferritin) (log_2_) μg/L* | 1.01 (0.91, 1.12); 0.87 | 1.15 (1.00, 1.32); 0.054 | 0.82 (0.70, 0.97); 0.023 | 0.002 |
|  |  |  |  |  |
| *Iron deficiency* |  |  |  |  |
| Ferritin <15 μg/L | 0.99 (0.74, 1.32); 0.95 | 0.79 (0.53, 1.17); 0.24 | 1.38 (0.89, 2.13); 0.15 | 0.04 |
| Ferritin ≥15 μg/L | Reference | Reference | Reference |  |
|  |  |  |  |  |
| **Preterm birth (<37 weeks)** |  | SPCQ (n=586) | SPAZ (n=622) |  |
| *Iron stores (measured by ferritin)* *(log_2_) μg/L* | 1.04 (0.89, 1.22); 0.61 | 1.06 (0.87, 1.30); 0.57 | 0.98 (0.75, 1.26); 0.85 | 0.74 |
|  |  |  |  |  |
| *Iron deficiency* |  |  |  |  |
| Ferritin <15 μg/L | 0.86 (0.56, 1.33); 0.50 | 0.80 (0.45, 1.41); 0.44 | 1.01 (0.51, 2.00); 0.98 | 0.79 |
| Ferritin ≥15 μg/L | Reference | Reference | Reference |  |
|  |  |  |  |  |
| **Small-for-gestational-age^1^** |  |  |  |  |
| *Iron stores (measured by ferritin)* *(log_2_) μg/L* | 1.11 (0.99, 1.23); 0.069 | 1.23 (1.05, 1.44); 0.010 | 0.99 (0.85, 1.15); 0.90 | 0.02 |
|  |  |  |  |  |
| *Iron deficiency* |  |  |  |  |
| Ferritin <15 μg/L | 0.93 (0.69, 1.24); 0.62 | 0.76 (0.50, 1.15); 0.19 | 1.14 (0.75, 1.72); 0.54 | 0.089 |
| Ferritin ≥15 μg/L | Reference | Reference | Reference |  |

**Note.** Linear regression analyses were performed for the outcome birthweight, with mean difference (95% CI) presented and logistic regression for the outcomes, low birthweight, preterm birth and small-for-gestational age, with Odds Ratios (95% CI) presented. Analyses were adjusted for gravidity, season, number of antenatal visits, clinic location, mid-upper arm circumference (< 23cm, ≥ 23 cm), stunting (height <150 cm, ≥ 150 cm), ethnicity, bed net use, smoking, betel nut use, and gestational age at enrolment, i.e. at ferritin measurement (as estimated by fundal height). Models also included the covariates sex of the newborn, timing of birthweight measurement (analyses including birthweight only), and treatment arm (overall analysis only).

Abbreviations: CI, confidence interval; OR, odds ratio; RC, regression correction; SPAZ, sulphadoxine-pyrimethamine plus azithromycin; SPCQ, SP plus chloroquine.

^1^ Defined as birthweight <10^th^ centile of the Intergrowth-21 reference [36].

**Table S3** Associations between maternal iron status (crude or adjusted ferritin) at first antenatal visit and pregnancy outcomes, stratified by gravidity. Madang Province, Papua New Guinea, 2009-2013 (n=1,832).

|  | **Gravidity** | |  |
| --- | --- | --- | --- |
|  | *Primigravida (n=899)*  **Adjusted mean difference, grams (95% CI); *p*** | *Multigravida (n=933)*  **Adjusted mean difference, grams (95% CI); *p*** | *p* interaction parameter |
| **Birthweight** |  |  |  |
| *Iron stores measured by ferritin (log 2) μg/L* |  |  |  |
| Ferritin | -23 (-46, -0.1); 0.049 | -3 (-25, 20); 0.82 | 0.32 |
| Adjusted ferritin | -20 (-43, 4); 0.098 | -4 (-27, 20); 0.76 | 0.39 |
|  |  |  |  |
| *Iron deficiency μg/L* |  |  |  |
| Ferritin <15 μg/L | 56 (-11, 123); 0.11 | -14 (-74, 46); 0.64 | 0.22 |
| Ferritin ≥15 μg/L | Reference | Reference |  |
|  |  |  |  |
| Adjusted ferritin <15 μg/L | 41 (-23, 107); 0.23 | 54 (-11; 119); 0.11 | 0.78 |
| Adjusted ferritin ≥15 μg/L | Reference | Reference |  |
|  |  |  |  |
| **Low birthweight (<2,500 g)** | **Adjusted OR (95% CI); *p*** | **Adjusted OR (95% CI); *p*** |  |
| *Iron stores measured by ferritin (log 2) μg/L* |  |  |  |
| Ferritin | 0.99 (0.87, 1.13); 0.90 | 1.06 (0.88, 1.28); 0.52 | 0.22 |
| Adjusted ferritin | 0.98 (0.86, 1.12); 0.79 | 1.06 (0.88, 1.28); 0.55 | 0.24 |
|  |  |  |  |
| *Iron deficiency μg/L* |  |  |  |
| Ferritin <15 μg/L | 1.01 (0.71, 1.45); 0.95 | 0.91 (0.55, 1.50); 0.71 | 0.33 |
| Ferritin ≥ 15 μg/L | Reference | Reference |  |
|  |  |  |  |
| Adjusted ferritin <15 μg/L | 0.93 (0.65, 1.34); 0.704 | 0.67 (0.40, 1.11); 0.121 | 0.106 |
| Adjusted ferritin ≥ 15 μg/L | Reference | Reference |  |
|  |  |  |  |
| **Preterm birth (<37 weeks)** | *Primigravida (n=588)* | *Multigravida (n=620)* |  |
| *Iron stores measured by ferritin (log 2) μg/L* |  |  |  |
| Ferritin | 1.07 (0.87, 1.31); 0.54 | 0.96 (0.74, 1.25); 0.78 | 0.75 |
| Adjusted ferritin | 1.02 (0.83, 1.26); 0.85 | 0.98 (0.75, 1.28); 0.89 | 0.99 |
|  |  |  |  |
| *Iron deficiency μg/L* |  |  |  |
| Ferritin <15 μg/L | 0.70 (0.38, 1.28); 0.25 | 1.15 (0.60, 2.20); 0.67 | 0.33 |
| Ferritin ≥ 15 μg/L | Reference | Reference |  |
|  |  |  |  |
| Adjusted ferritin <15 μg/L | 1.06 (0.57, 1.97); 0.86 | 0.70 (0.35, 1.40); 0.31 | 0.31 |
| Adjusted ferritin ≥ 15 μg/L | Reference | Reference |  |
|  |  |  |  |
| **Small-for-gestational-age^1^** |  | |  |
| *Iron stores (log_2_) μg/L* |  |  |  |
| Ferritin | 1.13 (0.98, 1.30); 0.090 | 1.11 (0.93, 1.32); 0.25 | 0.91 |
| Adjusted ferritin | 1.15 (0.99, 1.33); 0.066 | 1.10 (0.92, 1.31); 0.29 | 0.74 |
|  |  |  |  |
| *Iron deficiency* |  |  |  |
| Ferritin <15 µ/L | 0.92 (0.61, 1.37); 0.68 | 0.84 (0.54, 1.30); 0.43 | 0.79 |
| Ferritin ≥15 µ/L | Reference | Reference |  |
|  |  |  |  |
| Adjusted ferritin <15 μg/L | 0.77 (0.52, 1.14); 0.19 | 0.79 (0.49, 1.27); 0.33 | 0.98 |
| Adjusted ferritin ≥ 15 μg/L | Reference | Reference |  |

**Note.** Linear regression analyses were performed for the outcome birthweight, with mean difference (95% CI) presented and logistic regression for the outcomes, low birthweight, preterm birth and small-for-gestational age, with Odds Ratios (95% CI) presented. Analyses were adjusted for season, number of antenatal visits, clinic location, mid-upper arm circumference (< 23cm, ≥ 23 cm), stunting (height <150 cm, ≥ 150 cm), ethnicity, bed net use, smoking, betel nut use, and gestational age at enrolment, i.e. at ferritin measurement (as estimated by fundal height). Models also included the covariates sex of the newborn, timing of birthweight measurement (analyses including birthweight only), and treatment arm.

Abbreviations: CI, confidence interval; OR, odds ratio

Adjusted ferritin: adjusted for concomitant inflammation using the BRINDA (Biomarkers Reflecting Inflammation and Nutritional Determinants of Anemia) approach [16].

^1^ Defined as birthweight <10^th^ centile of the Intergrowth-21 reference [36].
